# Effects of head tilt on visual field testing with a head-mounted perimeter imo

**DOI:** 10.1371/journal.pone.0185240

**Published:** 2017-09-25

**Authors:** Sayaka Yamao, Chota Matsumoto, Hiroki Nomoto, Takuya Numata, Mariko Eura, Marika Yamashita, Shigeki Hashimoto, Sachiko Okuyama, Shinji Kimura, Kenzo Yamanaka, Yasutaka Chiba, Makoto Aihara, Yoshikazu Shimomura

**Affiliations:** 1 Department of Ophthalmology, Kindai University Faculty of Medicine, Osaka-Sayama City, Osaka, Japan; 2 CREWT Medical Systems, Inc., Tokyo, Japan; 3 Clinical Research Center, Kindai University Hospital, Osaka-Sayama City, Osaka, Japan; 4 Department of Ophthalmology, Graduate School of Medicine and Faculty of Medicine, The University of Tokyo, Tokyo, Japan; Tokai University, JAPAN

## Abstract

**Purpose:**

A newly developed head-mounted perimeter termed “imo” enables visual field (VF) testing without a fixed head position. Because the positional relationship between the subject’s head and the imo is fixed, the effects of head position changes on the test results are small compared with those obtained using a stationary perimeter. However, only ocular counter-roll (OCR) induced by head tilt might affect VF testing. To quantitatively reveal the effects of head tilt and OCR on the VF test results, we investigated the associations among the head-tilt angle, OCR amplitude and VF testing results.

**Subjects and methods:**

For 20 healthy subjects, we binocularly recorded static OCR (s-OCR) while tilting the subject’s head at an arbitrary angle ranging from 0° to 60° rightward or leftward in 10° increments. By monitoring iris patterns, we evaluated the s-OCR amplitude. We also performed blind spot detection while tilting the subject’s head by an arbitrary angle ranging from 0° to 50° rightward or leftward in 10° increments to calculate the angle by which the blind spot rotates because of head tilt.

**Results:**

The association between s-OCR amplitude and head-tilt angle showed a sinusoidal relationship. In blind spot detection, the blind spot rotated to the opposite direction of the head tilt, and the association between the rotation angle of the blind spot and the head-tilt angle also showed a sinusoidal relationship. The rotation angle of the blind spot was strongly correlated with the s-OCR amplitude (R^2^≥0.94, *p*<0.0001). A head tilt greater than 20° with imo causes interference between adjacent test areas.

**Conclusions:**

Both the s-OCR amplitude and the rotation angle of the blind spot were correlated with the head-tilt angle by sinusoidal regression. The rotated VF was correlated with the s-OCR amplitude. During perimetry using imo, the change in the subject’s head tilt should be limited to 20°.

## Introduction

A head-mounted perimeter termed “imo” (CREWT Medical Systems, Inc., Tokyo, Japan) is a newly developed portable standard automated perimeter (SAP) that was released in 2015 [1]. Because the imo is compact, it can be carried and used to test the visual field (VF) anywhere. Furthermore, using the imo, highly accurate measurement results can be obtained with examination times nearly equivalent to those of widely used SAPs such as the Humphrey Field Analyzer (HFA) (Carl Zeiss Meditec, Dublin, CA, USA) and Octopus perimeter (Haag-Streit, Koeniz, Switzerland).

Because the imo is head-mounted, subjects do not need to fix their head position during an examination. Thus, patients who could not previously participate in VF testing with a stationary perimeter might be able to undergo perimetry with the imo. Additionally, the positional relationship between the subject’s head and the imo is fixed; therefore, the effects of head position changes on VF measurements are small compared with those obtained with the stationary perimeter. However, only ocular counter-roll (OCR) induced by head tilt might affect VF testing.

When the head is tilted around the visual axis, a partially compensatory torsional eye movement occurs in the direction opposite to the head tilt. This eye movement is termed OCR. OCR is a compensatory vestibulo-ocular reflex (VOR) that stabilizes retinal images when the head is tilted [2]. OCR is classified into two types: dynamic and static. Dynamic OCR (d-OCR) is derived from both the semicircular canals and the otolith organs during active head roll movement [2–4]. Static OCR (s-OCR) is derived only from the otolith organs, particularly the utricles, which respond to gravitational direction changes while the head is kept tilted [2,4–9].

When the head is tilted during perimetry, the VF rotates, and the measurement point might interfere with adjacent test areas. Two factors are involved in this VF rotation: 1) the head-tilt angle and 2) the amount of OCR that is generated according to the head-tilt angle. To measure the VF more accurately, particularly when using the imo, we must clarify the relations among head tilt, OCRs and VF testing results, which have not been reported to date.

The purpose of our study was to quantitatively determine the effects of head tilt and OCRs on VF test results using the imo. Our hypothesis was that in perimetry with imo, the VF rotates through the OCR angle alone and toward the opposite direction from the head tilt because the head and the imo are always synchronized. To test the hypothesis, we observed OCRs and performed blind spot detection while subjects kept their head tilted at arbitrary angles. Based on our results, we determined the head-tilt angle that did not affect the VF test results.

## Subjects and methods

### A new head-mounted perimeter “imo”

An imo is equipped with two separate sets of optical systems and pupil-monitoring systems for the right and left eyes. Therefore, the imo can independently perform target presentations and pupil monitoring for either eye. This approach enables assessment of the VF not only monocularly but also binocularly at the same time (binocular random single-eye test).

A test target is displayed on a full high-definition (HD) transmissive liquid crystal display with high-intensity, light-emitting diode (LED) backlights. The surface of the target presentation is set 1 m away from the subject. The fixation target is set with a luminance of 8 cd/m^2^ (25.1 asb), a background luminance of 10 cd/m^2^ (31.4 asb), and a 0.5° visual angle.

The imo has an eye-tracking system to follow eye movement from the fixation point and correct the location of target presentation. For the eye-tracking system, the imo carries three near-infrared LED monitors with a wavelength of 950 nm. Images are recorded using an SXVGA resolution (1,280 × 960 pixels) complementary metal-oxide-semiconductor (CMOS) sensor with a maximum frame rate of 54 Hz.

The imo also carries a gravitational acceleration sensor and monitors the subject’s head position three-dimensionally in real time. The measurement accuracy is set to ±0.22°.

### Subjects

We studied 40 eyes of 20 healthy human subjects (11 males, 9 females; mean age, 33.7 ± 5.8 years; SE, right eye: -4.09 ± 2.64 D, left eye: -4.13 ± 2.82 D). The exclusion criteria were as follows: best corrected visual acuity < 1.0, refractive error greater than 7 D sphere, refractive error greater than 2.50 D cylinder, an ocular surgical history, ocular diseases that might cause VF loss, systemic diseases likely to affect the subject’s visual functions and fusion dysfunction because of strabismus. Furthermore, we examined the subjects’ history to confirm present and past histories of vestibular, neurologic or ophthalmologic diseases. Subjects with histories of those diseases were also excluded.

This cross-sectional study followed the tenets of the Declaration of Helsinki, and all the participants provided written informed consent after the ethics committee of Kindai University Faculty of Medicine (no. 26–239) approved the study.

### Methods

#### S-OCR measurement with head tilt

The imo can produce a head tilt toward different directions and at different velocities. However, it is considered that OCRs during typical VF testing consist mostly of s-OCRs because there is a lower incidence of rapid changes in the head position that alter torsional angular acceleration. Therefore, in this study, we observed s-OCRs.

We measured s-OCR amplitudes binocularly and head-tilt angles using a built-in gravitational acceleration sensor. Before each test, the subject’s head was kept upright for at least 15 sec. Next, the subject’s head was tilted toward a randomly selected head-tilt angle at a velocity of 2°/sec or lower. After reaching the target head-tilt angle, the head position was maintained at that angle. We made observations for 65 sec continuously in a single test. Head-tilt angles ranged from 0° to 60° to the right or left in 10° increments in one session of testing. During the test, the subject maintained central fixation as in conventional perimetry. All subjects underwent three sessions of tests, with each session performed on a separate day.

Eye movements were recorded in real time using the monitors for the eye-tracking system. The eye-tracking mode itself was not used. To track and measure torsional eye positions, we used a torsion method based on iris pattern recognition. In this method, the first frame was used as a reference. First, in the reference frame, we selected the two iris areas that were 1.56 mm apart from the pupillary edge, and the size was set to 1 mm × 1 mm. Second, the two iris areas were continuously tracked during recording. By comparing each recorded frame with the reference frame, the ocular torsion amount was computed by calculating the rotation angle of the two selected iris areas and by averaging the values. Third, we averaged all the ocular torsion values obtained at each targeted head-tilt angle. Then, we determined the average as the amplitude of the s-OCR. We defined the rotation of the eyes and head position in a clockwise direction from the subject’s point of view as positive rotation.

To avoid image processing errors of the apparatus, we eliminated data in which the three lights reflected from the three near-infrared LED monitors could not be identified and data in which the difference between the ocular torsion in two tracked iris areas in one eye was greater than 5°. Furthermore, we set aside the data obtained during the first 15 sec after setting the subject’s head at each head-tilt angle to minimize the effects of d-OCRs.

#### Blind spot detection with head tilting

To examine blind spot detection under the same conditions as those in s-OCR, we performed the binocular random single-eye test. Before each test, the subject’s head position was maintained at a targeted head-tilt angle for at least 15 sec. The head-tilt angles ranged from 0° to 50° to the right or left in 10° increments.

**Fig 1:**
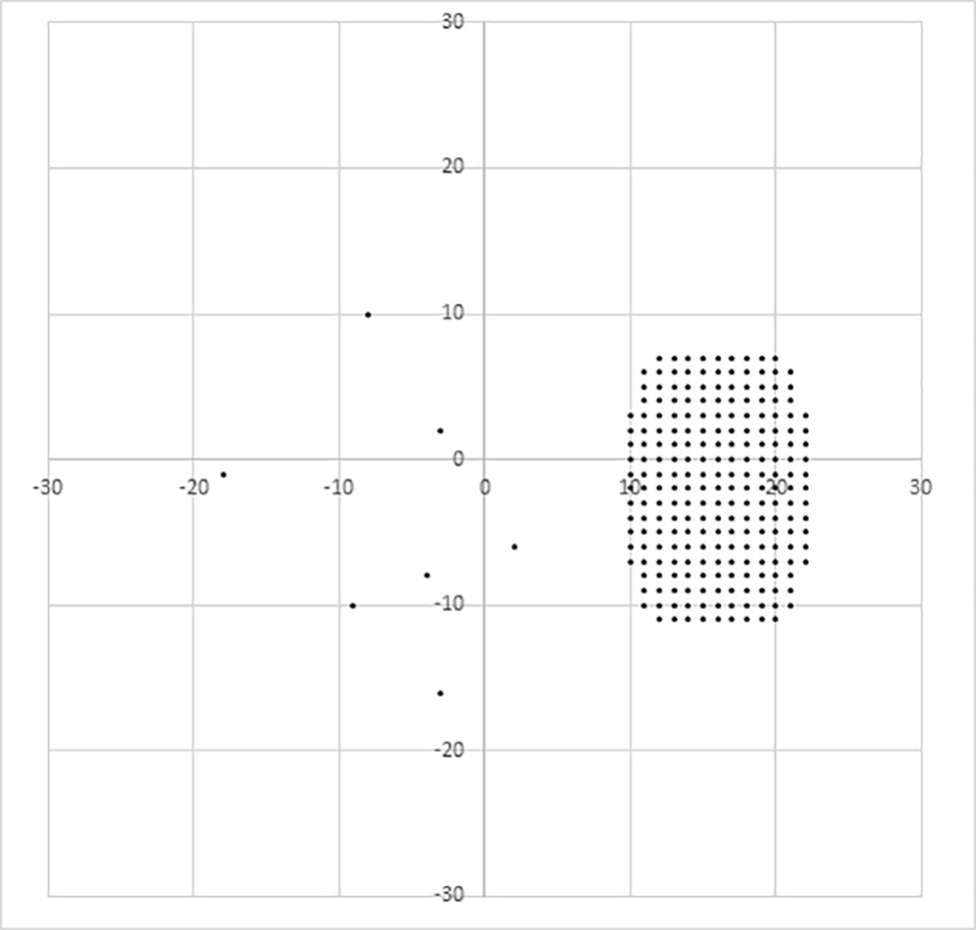
Blind spot detection program. Most of the 234 test points were arranged temporally within a 13° × 19° area with a central focus on (16°, -2°) in 1° steps. We determined that consecutive non-response areas represented the blind spot.

As a blind spot detection program, most of the 234 test points were arranged temporally within a 13° × 19° area with a central focus on (16°, -2°) in 1° steps (Fig 1). The test target luminance was a single light of 31.8 cd/m^2^ (100 asb), and the background luminance was 10 cd/m^2^ (31.4 asb). The target size was Goldmann size III (0.431° visual angle). The target presentation duration was 200 msec. The eye-tracking mode itself was not used.

Consecutive non-response areas of all 234 test points were considered to represent the blind spot. We defined the average of all the coordinates in the blind spot as the barycenter. Then, we calculated the difference between the barycenter angle in the vertical head position and in each head position. This difference was defined as the rotation angle of the blind spot. We considered the rotation of the eyes, head position and blind spot in a clockwise direction from the subject’s point of view as positive rotation.

We re-examined the tests when the rates of false positives and false negatives were greater than 3%, the blind spot was vague or the distance between the barycenter and fixation point differed by more than ±10% compared with the distance in the vertical head position.

### Statistical analysis

Regression analysis was performed to explore the relationships between the head-tilt angle and the amplitude of s-OCRs and between the head-tilt angle and the rotation angle of the blind spot. A mixed effects model [10] with subjects as a random effect was applied to take variance among subjects into account. In this analysis, linear and sinusoidal regression models with a 0 intercept were fitted, and Akaike’s information criterion (AIC) was used to determine the best fit. The coefficients of determination (R^2^) were also calculated. Furthermore, linear regression analysis with a 0 intercept was performed to explore the relationship between the amplitude of the s-OCR and the rotation angle of the blind spot. All statistical analyses were performed using JMP version 13.0 software (SAS Institute Inc., Cary, NC).

## Results

In all subjects, we observed torsional eye movement in the direction opposite to the head tilt while the subject kept their head tilted, i.e., s-OCR. In blind spot detection with head tilting, we detected rotation of the blind spot in the direction opposite to the head tilt in all subjects at all head-tilt angles.

### Head-tilt angle and amplitude of the s-OCR

**Fig 2:**
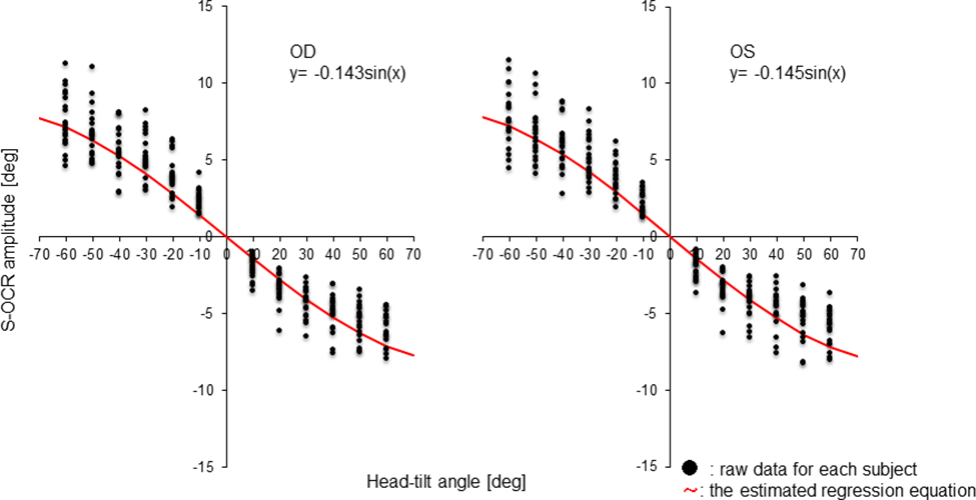
Relationship between head-tilt angle and S-OCR amplitude. The amplitude of the s-OCR tended to be large as the head-tilt angle increased in both eyes and in either direction of head tilting. This relationship was indicated by sinusoidal regression. The amplitudes of the s-OCRs differed among the subjects.

The amplitudes of the s-OCRs tended to be large as the head-tilt angle increased in both eyes and in either direction of head tilting. Table 1 summarizes the calculated values of the coefficient of determination and AIC, which revealed that the head-tilt angles and amplitudes of s-OCRs showed a better fit with sinusoidal regression than with linear regression in both eyes. Fig 2 shows all raw data for each subject and the estimated regression equation. The amplitudes of the s-OCRs differed among subjects. The respective regression coefficients were -0.143 and -0.145 in the right and left eyes, which suggested that the amplitudes of the s-OCRs at each head-tilt angle had nearly the same effect in both eyes.

**Table 1:** Coefficient of determination (R^2^) and Akaike’s information criterion (AIC) for linear and sinusoidal regression models.

	R^2^		AIC	
Ratio of head-tilt angle to:	Linear	Sinusoidal	Linear	Sinusoidal
Amplitude of s-OCR				
Right eye	0.93	0.94	-1013	-1055
Left eye	0.92	0.94	-1038	-1075
Rotation angle				
of the blind spot				
Right eye	0.91	0.91	-756	-774
Left eye	0.91	0.91	-775	-794

The gain of the s-OCR was defined as the ratio between the amplitude of the s-OCR and the head-tilt angle. The mean gain ± standard deviation (SD) values for 10°, 20°, 30°, 40°, 50° and 60° of head tilting to the right were 20±7%, 17±5%, 14±4%, 12±3%, 11±3% and 10±2%, respectively. The mean gain ± SD values of head tilt to the left were 22±7%, 19±6%, 17±5%, 15±4%, 13±3%, and 13±3%, respectively.

We further evaluated the respective inter- and intra-individual variabilities using between- and within-individual SDs. These values were calculated from the s-OCR values observed in three sessions of tests for all 20 individuals at every head-tilt angle. The results are shown in Table 2 for inter-individual variability and in Table 3 for intra-individual variability.

**Table 2:** Between-individual SDs at every head-tilt angle, indicating inter-individual variability.

Head-tilt angle	10°	20°	30°	40°	50°	60°
Right eye						
Tilt to the right	0.71	0.96	0.96	1.20	1.17	1.13
Tilt to the left	0.70	1.25	1.44	1.63	1.63	1.84
Left eye						
Tilt to the right	0.71	0.99	1.09	1.27	1.32	1.20
Tilt to the left	0.71	1.15	1.49	1.61	1.78	1.94

**Table 3:** Within-individual SDs at every head-tilt angle, indicating intra-individual variability.

Head-tilt angle	10°	20°	30°	40°	50°	60°
Right eye						
Tilt to the right	0.65	0.74	0.94	0.96	1.14	1.17
Tilt to the left	0.69	1.12	1.02	1.38	1.16	1.21
Left eye						
Tilt to the right	0.60	0.88	0.68	0.81	1.16	1.02
Tilt to the left	0.55	1.08	0.96	1.02	1.33	1.09

### Head-tilt angle and rotation angle of the blind spot

**Fig 3:**
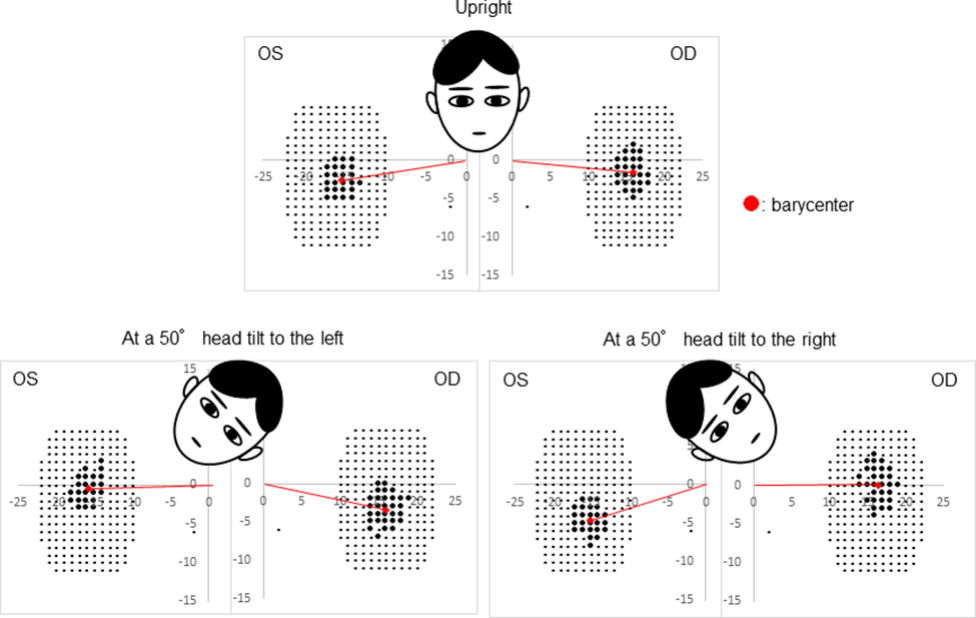
Blind spot detection results of a 33-year-old male subject. The barycenter of the blind spot rotated by head tilting. At a 50° head tilt to the right, the barycenter rotated in a counterclockwise direction. At a 50° head tilt to the left, the barycenter rotated in a clockwise direction.

Fig 3 shows the results of a 33-year-old male subject under the following conditions: the head position was upright and tilted 50° to the right and left. We detected the blind spot rotation in the direction opposite to head tilting. All subjects showed the same tendency.

**Fig 4:**
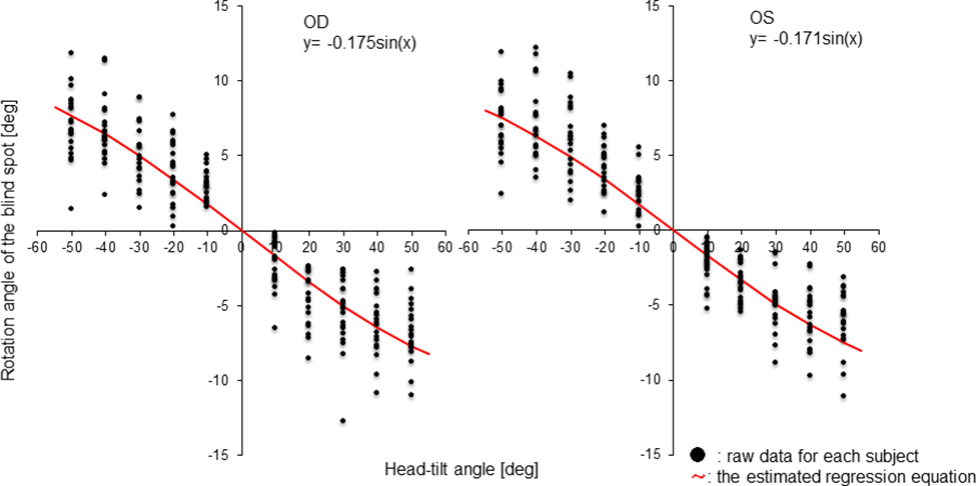
Relationship between head-tilt angle and rotation angle of the blind spot. The blind spot rotated in the direction opposite to the head tilt in all subjects at all head-tilt angles. The rotation angle of the blind spot tended to be large as the head-tilt angle increased in both eyes. This relationship showed sinusoidal regression.

The rotation angle of the blind spot tended to be large as the head-tilt angle increased in both eyes. As shown in Table 1, the head-tilt angle and the rotation angle of the blind spot also showed a better fit sinusoidal regression than with linear regression in both eyes. Fig 4 shows all the raw data for each subject and the estimated regression equation. The respective regression coefficients were -0.175 and -0.171 in the right and left eye.

### Amplitude of s-OCR and rotation angle of the blind spot

**Fig 5:**
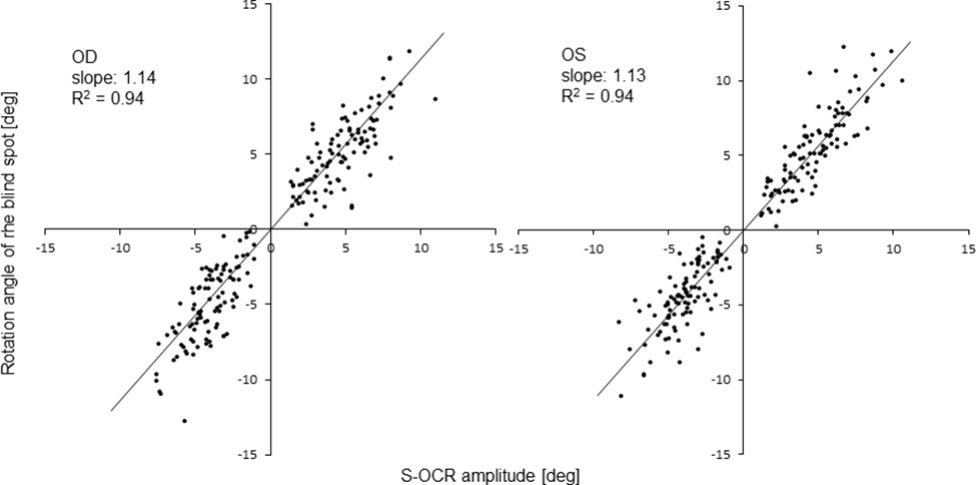
The correlation between S-OCR amplitude and rotation angle of the blind spot. These values were strongly correlated in both eyes (right eye: R^2^ = 0.94, *p*<0.0001, left eye: R^2^ = 0.94, *p*<0.0001), which strongly suggested that the rotated VF was correlated with the s-OCR amplitude in perimetry with imo.

Fig 5 shows the correlation between the amplitude of the s-OCR and the rotation angle of the blind spot in both eyes. These values were strongly correlated in both eyes (right eye: R^2^ = 0.94, *p*<0.0001, left eye: R^2^ = 0.94, *p*<0.0001).

### VF rotation and interference between adjacent test areas

When the VF rotates beyond the acceptable angle, the measurement point interferes with adjacent test areas. Table 4 summarizes the acceptable maximum VF rotation angle based on the degree of eccentricity and shows the head-tilt angle that generated the s-OCR amplitude equal to this acceptable maximum VF rotation angle. The head-tilt angle was calculated using the estimated regression equation shown in Fig 2.

**Table 4:** Acceptable maximum VF rotation angle based on the degree of eccentricity and the head-tilt angle that generated the S-OCR amplitude equal to the VF rotation angle.

The degrees of eccentricity					
30–2 (test points spaced at 6°)	3°	9°	15°	21°	27°
10–2 (test points spaced at 2°)	1°	3°	5°	7°	9°
Acceptable max. VF rotation angle	45°	18.4°	11.3°	8.13°	6.34°
Head-tilt angle	-	-	-	80°	50°

The acceptable maximum VF rotation angle does not lead to interference between adjacent test areas. The head-tilt angle that generated the acceptable maximum VF rotation angle was calculated using the estimated regression equation shown in Fig 2.

As shown in Table 4, the peripheral test areas, that is, at 27° eccentricity in the 30–2 pattern and at 9° eccentricity in the 10–2 pattern, were first affected by the VF rotation. The acceptable maximum VF rotation angle and the head-tilt angle at these peripheral test points were 6.34° and 50°, respectively. As shown in Fig 2, at a 50° head tilt, the amplitude of the s-OCR was greater than 6.34° in approximately half the subjects. At a 20° head tilt, the s-OCR was less than 6.34° in all subjects.

## Discussion

Our study showed that both the s-OCR amplitude and the rotation angle of the blind spot were correlated with the head-tilt angle by sinusoidal regression. Furthermore, we proved that the rotated VF was correlated with the amount of s-OCR in perimetry with imo.

During perimetry, subjects rarely tilt their heads rapidly. We therefore adopted a method for measuring s-OCRs while excluding the d-OCR as much as possible in this study. To be specific, we set the velocity of the head tilt to 2°/sec or lower [11] and randomly selected the head-tilt angle [6]. We also set aside the data obtained during the first 15 sec after setting the subject’s head at each head-tilt angle [6,12]. Additionally, we observed s-OCRs across three days with one test session per day. This strategy was employed for two reasons. The main reason is that we considered the effects of mounting or removing the imo on measurements of s-OCRs. We wished to verify the intra-individual variability caused by the effects of mounting and removing the imo. Testing on separate days means that the imo was mounted and removed on each day. The other reason is associated with the problem of measurement time. It has been reported that arousal of consciousness affects VOR [13] and that VOR has short-term adaptation [14]. Because of these findings, we wished to shorten the measurement time as much as possible.

The amplitude of the s-OCRs obtained from our study differed not only between individuals but also within the same individual. Tables 2 and 3 summarize the s-OCR variabilities and indicate the inter-individual variability and the intra-individual trial-to-trial variability, respectively. Indeed, both of the variabilities tended to increase as the head-tilt angle increased. We speculate that the inter-individual variability originated from the functional capability of the otolith organs or the torsional limitation of the extraocular muscles. Intra-individual variability might be normally observed and might be caused by the action of mounting or removing the imo.

Our study showed a sinusoidal relationship between the amplitude of the s-OCR and the head-tilt angle; this result is consistent with the results of previous reports [6,11,12,15,16]. The gain of s-OCRs found in our study is also in good agreement with previously reported values [6–9,17], although the methods used for the measurement and analysis of s-OCRs in our study differed from those used in previous studies. Our values showed the existence of limits on the amplitude of the s-OCRs and indicate that the compensation of the head-tilt angle amount by the s-OCR was low and decreased as the head-tilt angle increased. It is unclear whether these limitations are based on the normally limited torsional capacity of the extraocular muscles per se or on the limited responses of the otolith organs [8].

During the perimetry assessment, if the measurement point moves a distance equal to half the test location interval or more, that is, 3° in the test with a 6° test location interval and 1° in the test with a 2° test location interval, the measurement point interferes in adjacent test areas. Table 4 shows the acceptable maximum VF rotation angle based on the degree of eccentricity. The measurement points in the periphery are first affected by the VF rotation, as indicated by the value of 6.34° observed for a 50° head tilt. It is highly unlikely that the head position changes this value substantially during perimetry. However, as previously mentioned, the amplitude of the s-OCR differs substantially among individuals and even among the same individual. In fact, in this study, an s-OCR greater than 6.34° was found in approximately half of the subjects when the head was tilted at a 50° angle. At a 20° head tilt, the s-OCR was less than 6.34° in all subjects. At a 30° head tilt, the s-OCR exceeded 6.34° in more than 5% of the subjects. Based on these results, we suggest the existence of little interference between adjacent test areas when the subject’s head is tilted by less than 20° during perimetry with imo.

This study targeted healthy young subjects. In clinical practice, however, perimetry is mostly performed on glaucoma patients, who are typically elderly. S-OCR reflects the function of the otolith organs. However, this function declines with age [11,12]. Additionally, the amplitude of the s-OCR decreases with a decline in the function of the otolith organs. Thus, in clinical testing, the amplitude of the s-OCR might be lower than the amplitude measured in this study.

In conventional perimetry, however, the VF rotates with involvement of the head-tilt angle and the s-OCR amount. The portion of the head-tilt angle not compensated for by s-OCR would make the VF rotate in the same direction as the head tilt. Our results indicated that the maximum OCR compensation was approximately 20%. This low compensation by s-OCR indicates that VF rotation occurs in large part because of the head-tilt angle in conventional perimetry. The head fixation is, of course, a precondition in stationary perimetry; however, a small head-tilt angle amount compared with that observed in imo might cause interference between test areas.

Our study has two limitations. One is that the sample size was small. Considering the variabilities of s-OCRs, future studies should enroll more cases. The other is that we targeted only healthy, young subjects. In the future, we should also study cases from different age groups and with pathological VF, such as glaucoma.

To examine VF as accurately as possible, the OCR should be analyzed in real time with feedback for measurement of the VF. Indeed, we plan to correct eye torsion during VF testing using the eye-tracking system of the imo.

## Conclusions

Both the s-OCR amplitude and the rotation angle of the blind spot were correlated with the head-tilt angle by sinusoidal regression. The rotated VF was correlated with the s-OCR amplitude. During perimetry using imo, the change in the subject’s head tilt should be limited to 20°.

## References

[1] Matsumoto C, Yamao S, Nomoto H, Takada S, Okuyama S, Kimura S, et al Visual field testing with head-mounted perimeter ‘imo’. PLoS One. 2016;11(8):e0161974 doi: 10.1371/journal.pone.0161974 27564382 10.1371/journal.pone.0161974 PMC5001626

[2] Jorge Otero-Millan, Carolina Treviño, David S. Zee, John P. Carey, Amir Kheradmand. The video ocular counter-roll (vOCR): a clinical test to detect loss of otolith-ocular function. Acta Otolaryngol, (online), available from: http://dx.doi.org/10.1080/00016489.2016.1269364, (accessed 2017-03-01). 10.1080/00016489.2016.1269364 PMC5502765 28084887

[3] Schmid-Priscoveanu A, Straumann D, Kori AA. Torsional vestibuloocular reflex during whole-body oscillation in the upright and the supine position. I. Responses in healthy human subjects. Exp Brain Res. 2000;134(2):212–219. 11037288 10.1007/s002210000436

[4] Groen E, Bos JE, de Graaf B. Contribution of the otoliths to the human torsional vestibule-ocular reflex. J Vestib Res. 1999;9(1):27–36. 10334014

[5] Tarnutzer AA, Bockisch CJ, Straumann D. Head roll dependent variability of subjective visual vertical and ocular counterroll. Exp Brain Res. 2009;195:621–626. doi: 10.1007/s00221-009-1823-4 19415246 10.1007/s00221-009-1823-4

[6] Hamasaki I, Hasebe S, Ohtsuki H. Static ocular counterroll: Video-based analysis after minimizing the false-torsion factors. Jpn J Ophthalmol. 2005;49:497–504. doi: 10.1007/s10384-005-0254-4 16365796 10.1007/s10384-005-0254-4

[7] Ooi D, Cornell ED, Curthoys IS, Burgess AM, MacDougall HG. Convergence reduces ocular counterroll (OCR) during static roll-tilt. Vision Res. 2004;44:2825–2833. doi: 10.1016/j.visres.2004.06.014 15342226 10.1016/j.visres.2004.06.014

[8] Schworm HD, Ygge J, Pansell T, Lennerstrand G. Assessment of ocular counterroll during head tilt using binocular video oculography. Invest Ophthalmol Vis Sci. 2002;43:662–667. 11867581

[9] Bockisch CJ, Haslwanter T. Three-dimensional eye position during static roll and pitch in humans. Vision Res. 2001;41:2127–2137. 11403796 10.1016/s0042-6989(01)00094-3

[10] Laird NM, Ware JH. Random-effects models for longitudinal data. Biometrics. 1982;38(4):963–974. 7168798

[11] Diamond SG, Markham CH, Simpson NE, Curthoys IS. Binocular counterrolling in humans during dynamic rotation. Acta Otolaryngol. 1979;87:490–498. 313656 10.3109/00016487909126457

[12] Goltz HC, Mirabella G, Leung JCY, Blakeman AW, Colpa L, Abuhaleeqa K, et al Effects of age, viewing distance and target complexity on static ocular counterroll. Vision Res. 2009;49(14):1848–1852. doi: 10.1016/j.visres.2009.04.021 19409919 10.1016/j.visres.2009.04.021 PMC5104536

[13] Yagi T. Otolith function and eye movements. Equilibrium Res. 2005;64(4):258–261.

[14] Shutoh F, Ohki M, Kitazawa H, Itohara S, Nagao S. Memory trace of motor learning shifts transsynaptically from cerebellar cortex to nuclei for consolidation. Neuroscience. 2006;139:767–777. doi: 10.1016/j.neuroscience.2005.12.035 16458438 10.1016/j.neuroscience.2005.12.035

[15] Ohtsuki H. Memorandum on Bielschowsky Head Tilt Test in Superior Oblique Palsy. Jpn Orthoptic J. 2009;38:35–40.

[16] Diamond SG, Markham CH. Ocular counterrolling as a test of otolith function. Acta Otolaryngol. 1989;468:267–270. 2635516

[17] Pansell T, Tribukait A, Bolzani R, Schworm HD, Ygge J. Drift in ocular torsion during sustained head tilt. Strabismus. 2005;13:115–121. doi: 10.1080/09273970500216408 16251140 10.1080/09273970500216408

